# Progress in Medicine

**Published:** 1899-12-30

**Authors:** 


### Progress in Medicine.

### DISEASES OF THE HEART AND CIRCULATION.

**The Causation of Functional Heart-murmurs.**—In his Bradshaw lecture Foxwell[1] has ably discussed the causation of functional heart-murmurs, more especially of the well-known systolic pulmonary murmur. The clinical characteristics of this murmur are as follows: It is systolic in time, and never accompanied by a diastolic element; it is best heard in the second and third left intercostal spaces close to the sternum, and is best conducted upwards and outwards towards the left shoulder; it may reach the apex though losing greatly in loudness in doing so, and occasionally may be heard in the second right space. In character it is loud, harsh, and blowing, never loud and purring as in organic pulmonary stenosis. It is not fugitive, but remains with fair constancy until it vanishes for good. As a rule it is more evident in the supine than in the erect posture, especially if it be listened for immediately upon the patient's lying down. Accompanying this murmur we have the following signs: (*a*) The apex is pushed out and up, in a moderate instance lying beneath the lower part of the fourth space a little to the left of the nipple line. In the supine position this removal of the apex up and out is still further increased. Such apical displacement roughly corresponds to a movement along the arc of a circle the centre of which is the aortic valves, probably the most fixed portion of the heart. (*b*) The dulness is little, if at all, increased to the right. But the vertical dulness is much raised; instead of being at the upper border of the fourth costal cartilage at its junction with the sternum, it reaches to the middle of the third cartilage. This peculiar dulness is characteristic of the debilitated heart, and contrasts strongly with that of any heart enlarged by inflammatory disease of the pericardium or valves. (*c*) Another characteristic sign is the pulsation in the second, third, and fourth left spaces, which is evident and palpable, and extends from above downwards and outwards towards the apex. (*d*) Usually there is a slapping accentuation of the second sound; this often heralds the appearance of a murmur and lasts for some time after it is gone. It is evidently produced by a less degree of the same conditions which originate the murmur. (*e*) There is often, when lying supine, an indefinite pulsation in the veins in the right side of the neck; this disappears when the peripheral stream is cut off. (*f*) The pulse varies considerably, it is nearly always short and ends abruptly; the onset, too, is abrupt. The pulse wave has a trembling character, which is best described by the adjective "thrilly." An attempt is made to make up for this feebleness of effort by an increase of rate; it is rare for patients with pulmonary murmurs to have a pulse rate below 80. Of the symptoms which occur with this condition the chief and most evident is dyspnœa on exertion, especially if this exertion be in any way sudden. And no exertion can be long maintained; weariness, mental and physical, comes on very quickly. Palpitation, irritability, and dyspepsia are common. This pulmonary murmur very often occurs during adolescence, and is especially common in chlorotic girls. When it arises towards middle life it is most difficult to remove. The patients often give a history of cardiac strain, or the condition is a result of gradual lowering of the constitutional state.

In examining the heart after death, the most characteristic thing is the enlargement upward of the right ventricle; this dilatation is almost confined to the conus arteriosus. Moreover, the pulmonary artery is similarly widened. Here is evidently to be found the explanation of the increased vertical dulness to the left of the sternum, just as we find it on the right of the sternum in cases of aortic dilatation from chronic aortitis. The stretching of the conus carries the pulmonary valves upwards, so, that, on the average, they lie under the second costal cartilage. These are the chief conditions found during life and after death in cases of debilitated heart, and the explanation of the pulmonary murmur is the more likely to be the true one if it also explains the origin of the other conditions described. It is not due to the quality of the blood, for in many cases where this is greatly changed there is no murmur; again, the murmur frequently exists when there is no change in the blood. If the murmur were due to changes in the blood similar murmurs should arise in all parts of the body to a greater or less extent where there are angles or sharp curves. Immediately after severe hæmorrhage such generalised murmurs do arise, but they cease in a day or two, when the vessels are again sufficiently distended with (what must be) hydræmic blood. Also persons convalescing from exhausting diseases, *e.g.*, typhoid fever, whether suffering from anæmia or not, may have no murmur as long as they are confined to bed, but when sufficiently recovered to be up and about a murmur not seldom arises.

It has been suggested that in anæmia the corpuscles are lighter than normal, and so diverge from their usual axial position in the blood stream, and produce a murmur by their friction against the side of the vessel. But in anæmia the specific gravity of the blood plasma is also lessened, and such a theory would not account for the murmur in cases where there was no anæmia. It is therefore impossible that the murmur can be due to changes in the blood. It must arise, then, from some defect in the containing apparatus. It is not due to regurgitation through the mitral orifice; for its area of audition is very different from that of an organic mitral murmur, and the area of dulness is very different from that of mitral regurgitation. And similar reasons show it is not due to tricuspid leakage or aortic defect. Nor is it due to any lesion in the pulmonary valves, for these are, if death occurs, found to be healthy.

The cause of the murmur is to be found in the one post-mortem abnormality found in patients who have died with this functional pulmonary murmur—dilatation of the conus and pulmonary artery, with a carrying up of the root of the artery, so that in the supine condition it tends to lie over its bifurcation. There is never a diastolic murmur, hence the orifice of the valve cannot be dilated. We have, therefore, a tube with an annular constriction, a condition well known as able to induce a murmur. This annular constriction is the more effective since, owing to the carrying upwards of the pulmonary valve, the longitudinal strain is taken off the pulmonary artery, so that dilatation of that vessel is more likely to occur under the strain of the systolic inrush of blood. The cause of this dilatation of the conus of the right ventricle in debility is to be found in the strain which exertion, and especially sudden exertion, puts on the right side of the heart; and it is the upper end of the right ventricle which dilates, the lower part being restrained by one wall being formed by the septum ventriculorum, and held in check by the chordæ tendineæ of the tricuspid valve and their stout musculi. Further, the direction of the blood current is from the apex towards the pulmonary valves, and so the conus will tend to give where it is longest distended with blood. The dilatation of the pulmonary artery is probably secondary to the carrying of the pulmonary orifice upwards by the distending conus.

[1] Foxwell, Lancet, Nov. 4, 1899.

